# Advances in Nanomaterials Based on Cashew Nut Shell Liquid

**DOI:** 10.3390/nano13172486

**Published:** 2023-09-04

**Authors:** Ermelinda Bloise, Maria Rosaria Lazzoi, Lucia Mergola, Roberta Del Sole, Giuseppe Mele

**Affiliations:** 1 Department of Engineering for Innovation, University of Salento, Via Monteroni, 73100 Lecce, Italy; mariarosaria.lazzoi@unisalento.it (M.R.L.); lucia.mergola@unisalento.it (L.M.); roberta.delsole@unisalento.it (R.D.S.); 2 Institute of Atmospheric Sciences and Climate, ISAC-CNR, Str. Prv. Lecce-Monteroni km 1.2, 73100 Lecce, Italy

**Keywords:** CNSL, cardanol, anacardic acid, nanostructures, green chemistry, renewable materials

## Abstract

Cashew nut shell liquid (CNSL), obtained as a byproduct of the cashew industry, represents an important natural source of phenolic compounds, with important environmental benefits due to the large availability and low cost of the unique renewable starting material, that can be used as an alternative to synthetic substances in many industrial applications. The peculiarity of the functional groups of CNSL components, such as phenolic hydroxyl, the aromatic ring, acid functionality, and unsaturation(s) in the C_15_ alkyl side chain, permitted the design of interesting nanostructures. Cardanol (CA), anacardic acid (AA), and cardol (CD), opportunely isolated from CNSL, served as building blocks for generating an amazing class of nanomaterials with chemical, physical, and morphological properties that can be tuned in view of their applications, particularly focused on their bioactive properties.

## 1. Introduction

**Figure 1:**
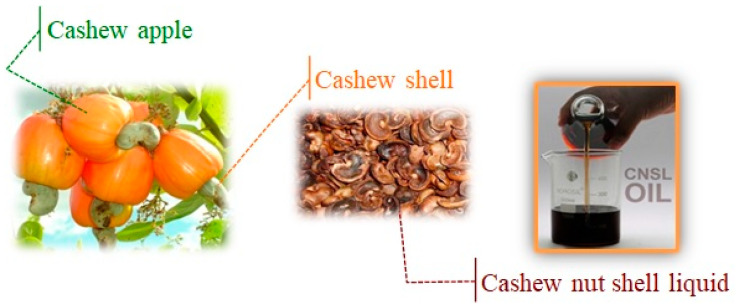
Cashew pedicel, fruit, and mesocarp filled with CNSL.

The global emergency of climate change is increasingly pressing and calls attention to action in every sector, such as food, energy, transport, and consumption in general. Hence, to meet the growing demand for chemicals, great efforts are being made to develop processes based on green chemistry principles, minimizing the use and generation of hazardous substances. In particular, the issue of agricultural waste disposal has stimulated several fields of interest, focusing on the potential transformation of refuse into valuable products. Green nanotechnology, intended as the combination of green chemistry principles and nanotechnologies, can be considered as an emerging field focused on exploring the potentiality and perspectives of green transformations and the formulation of nanomaterials. Increasing interest is devoted to this interdisciplinary science, especially for those case studies on use-oriented research approaching practical applications [1,2,3,4,5]. In this framework, new opportunities to develop alternative products by recovering new feedstocks from agro-industrial waste were started, and cashew nut shell liquid (CNSL) arose as a unique green precursor of the natural source of the long-chain phenolic compounds obtained as a byproduct of a cashew nut processing factory. In fact, CNSL is the oily greenish-yellow liquid filling the soft alveolar mesocarp shell of the cashew nut (Figure 1), which is the product of the cashew tree, *Anacardium occidentale* L., a popular plant in Brazil that is also present in many parts of the world [6].

For several years, many investigations have outlined a general overview of the extraction, isolation techniques, chemical composition, and physicochemical properties of CNSL extracts, exploring the different applications of their components and developing environmentally friendly protocols [6,7]. The CNSL classification and its composition, in relation to the extraction conditions, are well-known. It was established that anacardic acid (AA), cardanol (CA), cardol (CD), and 2-methylcardol are the four constituents: organic solvent extraction under mild conditions allows for obtaining a liquid rich in AA, the main component of so-called “natural CNSL”, while a high temperature produces the decarboxylation of AA, making CA the main constituent of the oil, called “technical CNSL”. When CNSL is treated with a high temperature (distillation at 180–200 °C), CA can be obtained at a >90% yield [6,8,9,10] (Table 1).

**Figure 2:**
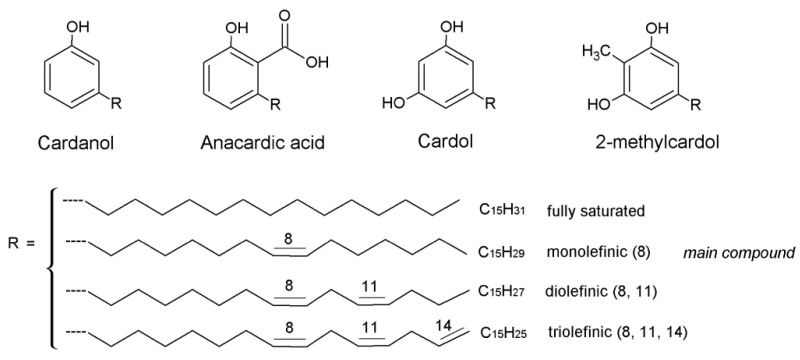
Constituents of CNSL.

The four CNSL components differ in their side chain unsaturation, as shown in Figure 2.

There are various sectors in which CNSL oil is applied: as a corrosion inhibitor for carbon steel, as a storage material, for transportation, in the manufacture of laminates to reduce brittleness and improve flexibility, as a substitute for linseed oil, as a binder in foundries, in automobile brake linings, and in dispersing and emulsifying agents, stabilizers, and hardening agents [8]. Hence, researchers have focused their attention on the most abundant constituents, AA and CA, and their different structure functionalities that make them convertible into useful industrial chemicals. Nevertheless, CA is highly exploited as it is the most abundant phenol in commercial-grade CNSL due to the facile decarboxylation of AA during the extraction, distillation, or refining processes of the crude extract. In addition to the phenolic hydroxyl group and nucleophilic aromatic carbons at the ortho- and para-positions, it has a peculiar long aliphatic side chain containing unsaturations as additional reactive sites that allow a great number of chemical modifications through safe operations, conferring hydrophobicity, low volatilization, and no aggressive odor. Some examples of structural modifications made to the hydroxyl group are the reaction with acid chlorides in the presence of alkalis or bases to give the corresponding cardanyl esters, the etherification via the reaction with various alkyl halides, the sulfonation to produce alkylaryl sulphonic acids or their metal salts, and, again, the epoxidation via the reaction with epichlorohydrin in the presence of caustic soda as a catalyst. In particular, the chemical versatility of CNSL has made it possible to achieve many polymeric products using several techniques with extraordinary flexibility and a hydrophobic character due to the “internal plasticization” of the long side chain [9]. In addition to fine chemicals, functional molecules, and polymers, liquids from cashew shells have attracted the attention of pharmaceutical research, studies from which have reported different biological activities, such as antioxidant, anti-inflammatory, antidiarrheal, anticancer, antimicrobial, and antimutagenic ones, important for pharmaceutical and cosmetic formulations [9,10].

This review focuses on and explores the last fifteen years of progress in the use of CNSL and its components as renewable raw materials. The intent is to highlight the valorization of its chemical and biological versatility through nanotechnologies, discussing selected examples of a wide range of functional nanomaterials for several applications. In particular, we focused our attention on nanostructures obtained from CA and AA, which are the most studied components of CNSL, to provide a potential platform for the preparation of nanostructured systems via the conversion of agricultural waste into valuable products.

## 2. CNSL-Based Nanomaterials

Exploiting the oily nature of CNSL, which makes it poorly soluble in water, some studies have used the phenolic mixture just as it is via a nanotechnological approach to scientifically validate a new form of a nanoemulsified tool in different fields of application. Al-Hazzani et al. [11] developed and evaluated the in vitro cytotoxic activity of a CNSL-based nanoemulsion against human breast cancer cells (MCF-7), a major female cancer that is mainly treated with painful and toxic techniques, such as surgery, chemotherapy, and radiotherapy. In this study, a stable oil-in-water nanoemulsion, with droplets ranging from 400 to 1000 nm, was obtained by emulsifying fine CNSL (1%) with a cell culture medium in the presence of Polysorbate 80, a nonionic hydrophilic surfactant. The effects of the nanoemulsion treatment on cell viability were investigated via an MTT assay, indicating a dose- and time-dependent inhibition of tumor cell growth. In fact, the IC_50_ value (i.e., the CNSL nanoemulsion concentration necessary to inhibit the growth of 50% of tumor cells) was higher for a 24 h treatment, i.e., 140 ± 10.5 µL/mL, while for cells treated for 48 h the IC_50_ value was in the range of 88 ± 14.2 µL/mL. Morphological studies of treated cells revealed that the nanoemulsion induces cytological changes that cause cell death through apoptosis and necrosis mechanisms. This preliminary study confirms that a CNSL nanoemulsion has shown cytotoxic and growth-inhibiting effects on MCF-7 cells, thus providing a scientific basis for its anticancer properties with respect to breast cancer.

**Figure 3:**
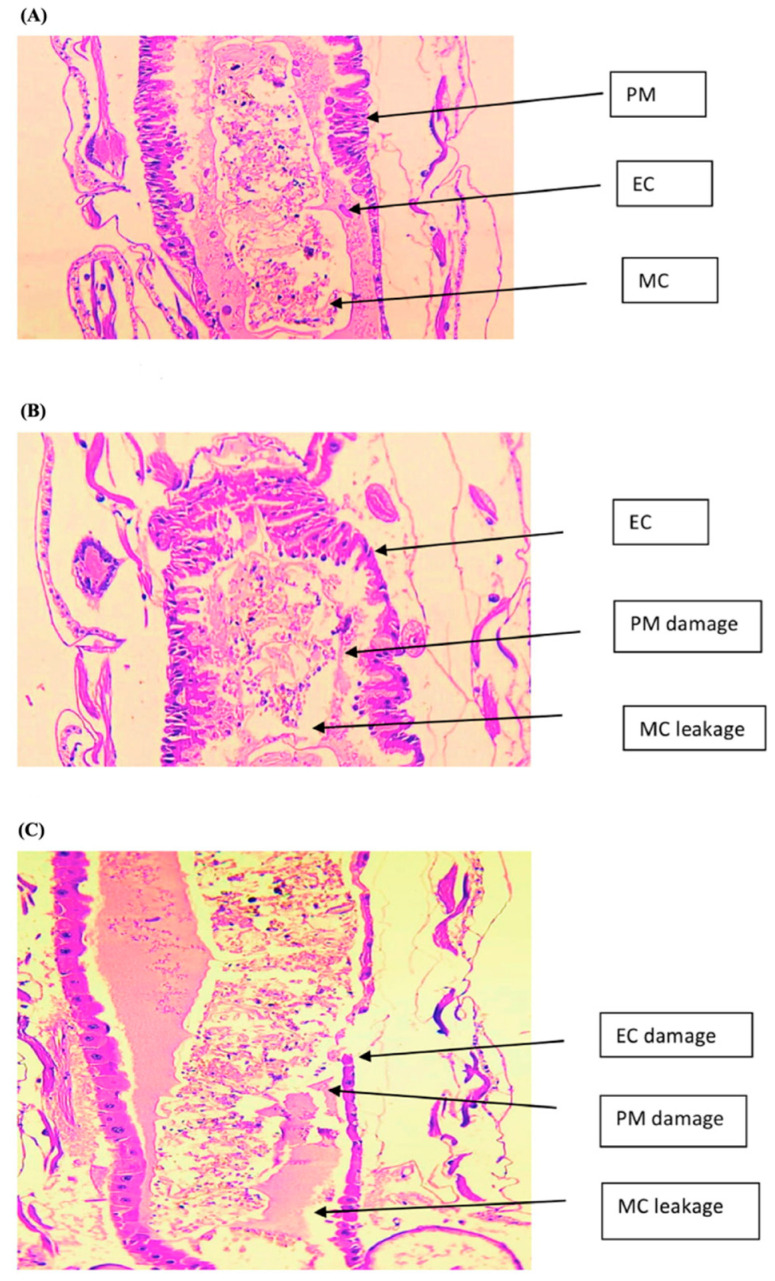
LS of larvae treated, showing the midgut region epithelial cells (ECs), peritrophic membrane (PM), and midgut content (MC) of (**A**) control, (**B**) LC_50_ (24 h) of bulk CNSL, and (**C**) LC_50_ (24 h) of nano-CNSL observed at 100× magnification. Reproduced from ref. [12], Elsevier, 2019.

The right combination of nonionic surfactants and propylene glycol as a cosurfactant can produce monodispersed micelles of CNSL with a lower dimension (mean diameter of 52 nm) that are stable even after storage for 2 months at 25 °C and 45 °C [12]. The bioefficacy of this nanoformulation was investigated as a plant-based waste nanopesticide against the malaria vector *Anopheles culicifacies*, and compared with its bulk emulsion form. Morphological observations and histopathological alterations were evaluated to understand the possible action mechanism of the CNSL nanoemulsion against the larvae. Larval mortality, evaluated as lethal concentration (LC_50_), was dose- and time-dependent, with values of 18.1 mg/L and 1.4 mg/L for bulk and nano-CNSL, respectively. The SEM examination revealed the uptake and impregnation with nanoemulsion droplets through the cuticle of the larva, completely deformed by the nano-CNSL treatment. A thin longitudinal section (LS) was examined under the microscope (Figure 3) to evaluate the histopathological impact on treated *Anopheles* larvae with LC_50_.

Nanoemulsion-treated larval tissues displayed significant damaged and broken epithelial cells and peritrophic membranes with extensive midgut content leakage. This CNSL nanoemulsion combines pesticidal action with the effective utilization of waste, significantly reducing the pesticide load and toxicity to the environment, while enhancing larvicidal activity compared to bulk CNSL. Furthermore, it could be applied to controlling the immature stages of mosquitoes in breeding sites such as stagnant water sources.

Another approach consists of the chemical modification of tCNSL as a sulfonate and using it as an ionic surfactant for both tCNSL and CA to form O/W nanoemulsions such as larvicidal and antimicrobial agents for controlling mosquitoes of *Aedes aegypti* [13]. Although sulphonate derivatives did not show antimicrobial and larvicidal activities, they conferred solubility and detergency when mixed with CA and tCNSL, allowing the formulation of emulsions with larvicidal and surfactant activity for effective dispersion in an aquatic environment. Concerning the larvicidal activity in *Ae. aegypti* and solubility, the smallest micelles (136.7 ± 3.9 nm), obtained with a tCNSL + sodium-tCNSL-sulphonate emulsion mixture, presented higher performance compared to the analogue with CA, probably due to easier penetration into cells causing cellular damage, resulting in apoptosis/necrosis. Therefore, the nanoemulsion represents an effective multifunctional larvicidal and surfactant product that is highly available, although further detailed analysis of the mixture composition is needed, which depends on the heat treatment and origin of the cashew nut, as well as ecotoxicological tests, to ensure an environmentally safe product.

CNSL has also been used as a natural binder or chemically modified resin to develop antibacterial nanostructured films for eco-packaging materials, surface coatings, adsorption, and water purification. Bioformulated nanosheets with CNSL, iron oxide nanoparticles, chitosan, and plant extracts, obtained via the solution casting method, showed oxygen barrier properties as well as thermal stability, microbial resistance, and UV resistance [14]. Characterization studies show a uniform distribution of CNSL and iron oxide nanoparticles (average size of ~100 nm) on a chitosan matrix with mixtures of bioactive compounds. The nanosheets appear as multiphase materials with a dense, compact structural formation. The incorporation of CNSL acts as a natural phenolic binder to provide strong interactions between active biocompounds and the chitosan matrix, while the embedded iron oxide nanoparticles intertwine with CNSL, resulting in a material with enhanced thermal stability. Additionally, they have the potential to inhibit UV absorption, a useful property for packaging materials for oxygen-sensitive food products. Evaluation tests of microbial resistivity to *E. coli*, *B. subtilis*, *P. aeruginosa,* and *S. aureus* microorganisms on nanosheets imbibed in water showed excellent antimicrobial resistance properties. Combining CNSL with iron oxide nanoparticles results in an improvement of the hydrophobic properties and also the inhibition of bacteria growth on the surface, which makes them commercially usable for multifaceted packaging applications in the food industry.

Zafar et al. [15] report the production of nanostructured freestanding polymeric films/coatings by using an innovative, widely applicable, rapid, cost-effective, and environmentally friendly green approach. The metal–organic framework, achieved through the coordination of methylolated-CNSL and Mg (II) ions (Mg(II)CNSL), was cured via reaction with aliphatic amine (FA) to produce nanostructured [Mg(II)CNSL-FA], confirmed through FTIR, ATR, XRD, SEM/TEM, and DSC studies. The inclusion of Mg(II) in CNSL-FA directed various properties, in particular a fireproofing feature, superior thermal stability (~300 °C), and a hydrophobic nature, explained by the formation of a semicrystalline three-dimensional structure with the stacking of nanorods (24.7 nm × 3.4 nm) coordinated with Mg(II) ions. Antibacterial activity was tested against *S. aureus, B. subtilis, E. coli,* and *P. aeruginosa*. The films were inactive against *P. aeruginosa*, while a higher percentage of inhibition, especially for *B. subtilis* and *E. coli,* could be attributed to the disruption of membrane integrity due to electrostatic interactions occurring between positively charged ions in Mg(II)CNSL-FA and negatively charged lipids on the bacterial surface.

### 2.1. Cardanol-Based Nanomaterials

In this section, we report the most relevant studies on nanomaterials made starting from CA, the most abundant and explored component of the tCNSL and CNSL mixture. Some examples of CA-based metal nanomaterials have been produced via coordination polymerization of the phenolic compound with metal ions or molecules derived from chemical modifications of CA that have shown very versatile properties (optical, photophysical, electrochemical, etc.) and are used to prepare composite nanomaterials with metal oxides. A promising approach, applied to water purification, is heterogeneous photocatalysis using nanostructured semiconductors. In this context, nanocomposite materials based on ZnO nanostructures, impregnated with CA-derived lipophilic porphyrins (H_2_Pp-metal-free and CuPp-copper porphyrin), provide an alternative technology to efficiently remove toxic substances from water under environmental conditions [16]. In this way, nanomaterials with a diameter of 55 nm were obtained. Moreover, FTIR studies confirm the noncovalent nature of the interactions between CA-porphyrins and ZnO. The photocatalytic activity was investigated via the degradation of rhodamine B (RhB) in an aqueous solution under visible-light irradiation and natural sunlight. Porphyrins are photosensitizing agents for semiconductors; thus, the composite nanomaterials showed better absorption in the visible region than bare ZnO did. Moreover, the pivotal role played by the peripheral substituent of porphyrins has been demonstrated, which, because of their length and flexibility (from CA), contribute to improving the charge transfer to ZnO. The presence of porphyrins increases the recombination times of charge carriers photogenerated in the ZnO, also confirmed by photoluminescence. The photodegradation test of RhB promoted by sunlight allowed the establishment of the positive effect of the incident light intensity and a cooperative mechanism involving the UV component, demonstrating the photocatalytic efficacy of composite nanomaterials with porphyrins derived from renewable materials.

An advanced functional material, like a metal–organic framework (MOF), was produced via the microwave-assisted synthesis of a renewable organic ligand CA and nontoxic endogenous cation Mn(II) bivalent salt [17]. The synthesis was carried out by a “solvent-free in situ” approach, and FTIR spectroscopy was used to confirm the structure and verify the curing of the material (MnIIMicCol). The morphological characterization of the nanomaterial investigated through XRD, optical microscopy, SEM, and TEM showed an amorphous and layered morphology and mesoporous (pore diameter of 8.0286 nm) behavior. The thermal behavior measured by the TGA/DTG/DSC techniques confirmed a high inherent thermal stability. Antibacterial activity was tested against Gram-negative (*E. coli* and *K. pneumoniae*) and Gram-positive (*B. subtilis* and *S. aureus*) bacterial strains, revealing two bactericidal mechanisms: (i) damage to the bacterial cell membrane and (ii) the production of reactive oxygen species (ROS), which may cause oxidative stress on bacteria cells and damage to both DNA and RNA. The excellent chemical–physical properties and the moderate antibacterial activity make this nanostructured MOF usable in thermally stable (up to 230–250 °C) antimicrobial coating materials.

**Figure 4:**
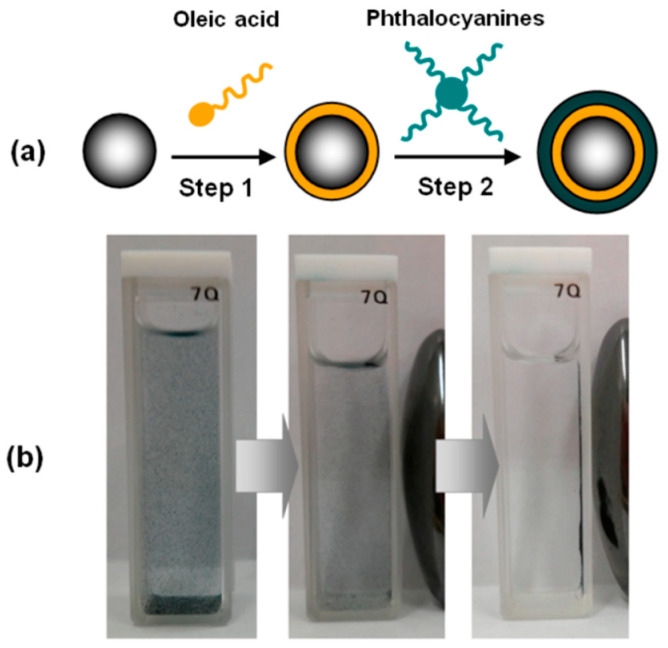
(**a**) Schematic representation of preparation of magnetic phthalocyanines: step (1) ferrofluid produced with a mixture of Fe_3_O_4_ and oleic acid; step (2) reaction of ferrofluid with phthalocyanines. (**b**) Influence of the magnetic field under the suspension of Fe_3_O_4_@OA/Pc in ethanol. Reproduced from ref. [18], MDPI, 2019.

Ribeiro et al. [18] reported the preparation of hybrid nanomaterials in a core/shell/shell morphology with an average diameter of 12–15 nm, formed by a magnetite core Fe_3_O_4_ and two layers that were oleic acid (OA)- and phthalocyanine CA-based, with different transition metals coordinated with phthalocyanine centers (Fe_3_O_4_@OA/Pc, Fe_3_O_4_@OA/CoPc, Fe_3_O_4_@OA/CuPc, Fe_3_O_4_@OA/NiPc, and Fe_3_O_4_@OA/ZnPc) (Figure 4).

The aim was to form potential therapeutic agents for photodynamic therapy and for magnetic hyperthermia, a promising therapeutic approach for cancer treatment for which Fe_3_O_4_ nanoparticles have shown excellent properties. Fe_3_O_4_@OA/Pcs has decomposition temperatures of 380 °C, which give excellent stability, which is affected by the presence of different transition metals in the cavity of the phthalocyanine. The stability and magnetic properties of these hybrid nanomaterials are due to the Van der Waals interactions established between the phthalocyanines and alkyl chains of oleic acid emerging from the surface of Fe_3_O_4_, while the transition metals of metallo-Pcs negatively influence the magnitude of the nanomaterial magnetization.

In some studies, CA has been used as a polymer matrix for the preparation of nano-biocomposites. A prepolymer (resol) was synthesized by mixing CA with an aqueous solution of formaldehyde, which was then mixed with epoxy resin as a modifier to prepare a thermosetting plastic. The polymer was mixed with spongy gourd fiber as a mechanical strengthening agent, and with different amounts of magnetite nanoparticles, which conferred specific superparamagnetism properties [19]. After complete homogenization, a crosslinking agent was added and submitted to the curing process. All of the nano-biocomposites had superparamagnetic characteristics, good biodegradation rates in simulated soil, and excellent thermal stability. The addition of magnetite nanoparticles improved the mechanical strength. All of the materials were tested to evaluate their potential applications as dielectric resonators. The range of frequency in which the antenna presents an effective performance (BW) for all of the nano-biocomposites was verified in −10 dB in the return loss graph, and it is not affected by magnetic nanoparticle content, making them suitable for technological applications, especially for broadband performance.

In newer research, sheet-like nano-biocomposites, based on CA thermosetting resin [20], were obtained. The inclusion of cellulose nanofibrils and nanoplatelets of expanded graphite improved in synergy the flammability, thermal, mechanical, and water absorption properties of nano-biocomposites, required for applicability in coating systems and automotive applications, where weight reduction and a reduction in VOCs in the environment are of great importance. The assembly of this composite material allowed for improving the dispersion of nanofiller cellulose nanofibrils with a high specific surface area and a high percentage of exposed atoms on their surfaces. Furthermore, the CA resin showed a stabilizing effect from the expanded graphite in the nanosheets.

The versatile behavior of CA and its derivative small molecules were found to be attractive in functional soft nanomaterials research to generate self-assembled morphologies down to 100 nm dimensions, such as nanotubes, nanofibers, gels, surfactants, and liquid crystals [21,22]. Gels are systems delicately balanced between molecules’ precipitation and solubilization in a solvent that, self-assembling through noncovalent interactions, form a fibrous network that traps the solvent through capillary forces and resists the flow of the medium. Pyrene-coupled coumarin derivatives with varying hydrophobic chains have been synthesized via aldol condensation, starting from CA-aldehyde, obtained through electrophilic aromatic substitution reactions [23]. The formation of transparent fluorescent organogels occurred via supramolecular self-assembly through the π–π stacking of pyrene units and hydrogen bonding. In particular, absorbance and emission and ^1^HNMR studies showed that hydrogen bonding between carbonyl groups of coumarin coupled pyrene with the hydroxyl group of a solvent, and π–π stacking interactions have driven the self-aggregation and gel formation processes. The presence of saturated and unsaturated hydrophobic tails affects the gelation efficiency tested in different solvents, strongly influencing the optical properties of π-conjugated derivatives. From these results, self-assembly nanoflakes were derived, and in vitro fluorescence imaging reveals that these compounds inhibit the proliferation of PC3 prostate cancer cells, making them potentially applicable in the cell imaging field. Another study reported the synthesis of coumarin-tris-based amphiphiles, which in turn have been derived from CA [24]. This small amphiphilic system showed the ability to form a stable supramolecular hydrogel sensitive to external stimuli such as pH or the presence of the biologically important Fe^3+^ ion. Optical microscopy and high-resolution transmission electron microscopy (HRTEM) investigations revealed a reversible morphological transition from self-assembled gels at neutral and basic pH levels to vesicles and nanotubes when pH is acidic. ^1^HNMR and XRD studies suggested that the π–π stacking interactions and hydrogen bonding were the driving forces for the gelation process. Moreover, the release of the chemopreventive drug curcumin encapsulated in the gel has been driven by a gel-to-sol transition induced by pH and Fe^3+^ metal ion stimuli, providing a potential stimuli-responsive drug delivery system for in vivo formulations.

Several studies have involved the use of CA derivatives as dopants to achieve polyaniline nanomaterials, which have attracted wide interest due to their unique electrochemical properties and processability in electronic and optical devices. For example, CA was used to synthesize 4-[4-hydroxy-2((Z)-pentadec-8-enyl)phenylazo]benzenesulfonic acid, an amphiphilic molecule that acts as a doping agent in a unique micellar soft template approach. Through a selective templating process in water [25], various types of polyaniline nanomaterials (fibers, rods, spheres, and tubes) have been developed. The process can be carried out in a controlled way, depending on how the starting materials (amphiphilic dopant, aniline, ammonium persulfate, and water) are self-organized and polymerized in water via emulsion, dilution, and interfacial. DLS, TEM, and X-ray diffraction measurements displayed the fact that the amphiphilic dopant plays a key role during the chemical oxidative polymerization of aniline, forming thermodynamically stable aggregates of inherent nanoscale dimensions in a solution, which act as templates for the overall morphology of the resulting polyaniline. Cylindrical micelles or bilayers with an aniline monomer were obtained via the emulsion route, and their oxidation yields nanofibers and nanotubes, respectively. With the dilution, the emulsion was transformed into micelle aggregates of 175 nm size, which are seeds for nanorods. In the interfacial route, the spherical aggregate formed by the dopant–ammonium persulfate complex acts as a template for polyaniline nanospheres of 200–400 nm. Further studies confirmed how the amphiphilic dopant structure and polymerization pathways played an important role in the morphology, solid-state ordering, and bulk conductivity of polyaniline nanomaterials [26,27]. Even for (3-dodecyl-8-enylphenyloxy) butane sulfonic acid, synthesized via the ring opening of butanesultone with CA under basic conditions [28], the authors studied its successful use as a structure-directing agent for polyaniline nanomaterials via the emulsion and dispersion routes. In the emulsion route, the cylindrical micellar soft template, formed between the dopant and aniline, leads to the formation of nanofibers, whereas in the dispersion route the vesicular template of aniline + toluene produces nanotapes, both highly soluble, and therefore suitable for various applications in bio- and chemical sensors, as well as optical devices. Balachandran et al. [29] realized an amphiphilic building block, N-cardanyl tauramide, a biobased surfactant obtained via the judicious combination of CA as the hydrophobic part and taurine (a vital aminosulfonic acid) as the hydrophilic head group. Cryoelectron microscopic measurements and other physicochemical techniques were used to investigate vesicle formation and vesicular adhesion, showing an adaptive, responsive behavior of unsaturated alkyl chains present in the CA-based amphiphile. A reversible micelle-to-vesicle transition, followed by vesicular adhesion leading to a sticky jelly phase, was observed to be temperature-responsive.

**Figure 5:**
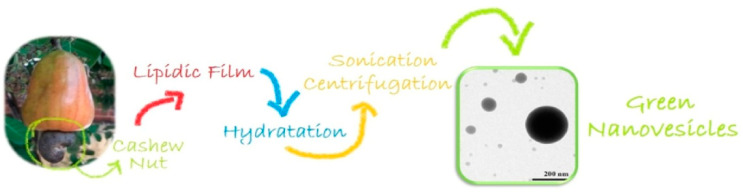
Solvent-free batch method for the preparation of CNSL-based nanovesicles.

In recent years, increasing attention has been directed to the field of green chemistry, which has strongly motivated researchers to develop and design new biobased soft materials. The structural uniqueness of the CA molecule allows it to show surface activity in particular conditions, even when not chemically modified. Therefore, some researchers have used CA as a molecular building block for the practical and environmentally friendly batch preparation of nanovesicle classes, in view of their potential applications in the pharmaceutical and biomedical fields. Organic solvent-free synthetic pathways (Figure 5) were developed in which CA and cholesterol (CH) mixtures have been used to embed minor amounts of functional molecules inside nanometric-sized vesicles (from 100 to 300 mn). Specifically, nanovesicles hosting effectively both hydrophobic and hydrophilic bioactive molecules, such as a porphyrin–cardanol hybrid and chlorogenic acids, were prepared [30,31].

Considering the versatility of these CA-based nanomaterials to load molecules of different chemical natures, four various phthalazines derivatives have been embedded in nanovesicles, and their biological activities against different cancer cell lines [32] showed that CA itself confers good antioxidant and moderate cytotoxic properties to unloaded nanovesicles, which have been improved via loading with some phthalazine molecules.

The adaptability of this nanosystem allowed it to incorporate cannabidiol [33], a cannabis extract compound known for its intrinsically low chemical stability, which limits its therapeutic potential. The use of nitrogen gas during the synthesis provided the inert atmosphere required to avoid the thermooxidative degradation of cannabidiol. Stability studies showed that the embedded cannabidiol structure was also preserved because of the antioxidant properties of CA for 30 days when stored at 20 °C.

**Figure 6:**
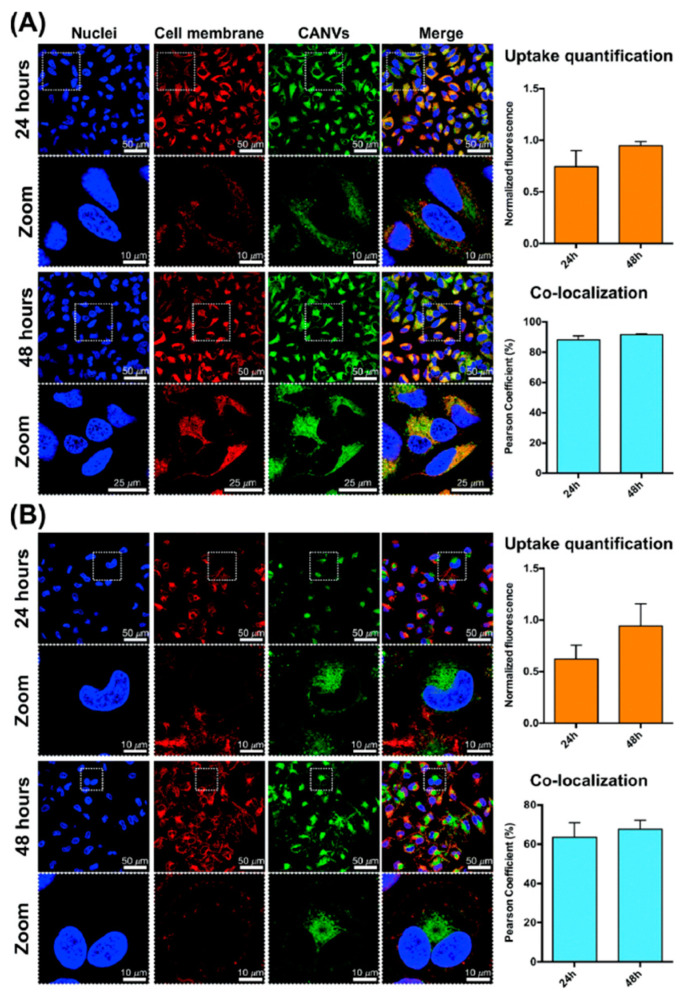
Uptake study of fluorescent CA nanovesicles in HeLa cells (**A**) and human macrophages (**B**) after 24 and 48 h of incubation. The dashed squares are higher-magnification images of the selected area. Uptake quantification and co-localization studies are reported on the right-hand side. Reproduced from ref. [34], Royal Society of Chemistry, 2019.

RhB, as a fluorescent reporter, was efficiently encapsulated in CA-based nanovesicles in order to develop a green system for imaging biological samples [34]. These fluorescent nanovesicles can be delivered to human macrophages and HeLa cells, which show an efficient active uptake process (Figure 6), and their biocompatibility up to specific concentrations was confirmed.

**Figure 7:**
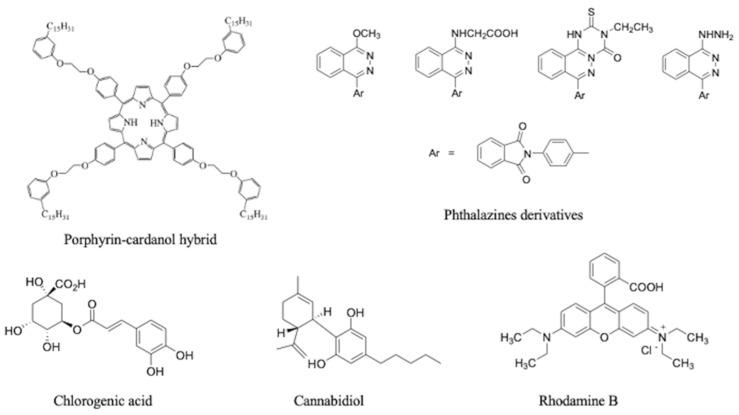
Active molecules encapsulated in CA-based nanovesicles via the solvent-free batch method (porphyrin–cardanol hybrid [30], phthalazine derivatives [32], chlorogenic acid [31], cannabidiol [33], and rhodamine B [34]).

Figure 7 summarizes the five kinds of active molecules incorporated into CA nanovesicles via the solvent-free batch method, and used as a platform to develop the green and bioactive nanosystems described so far.

**Figure 8:**
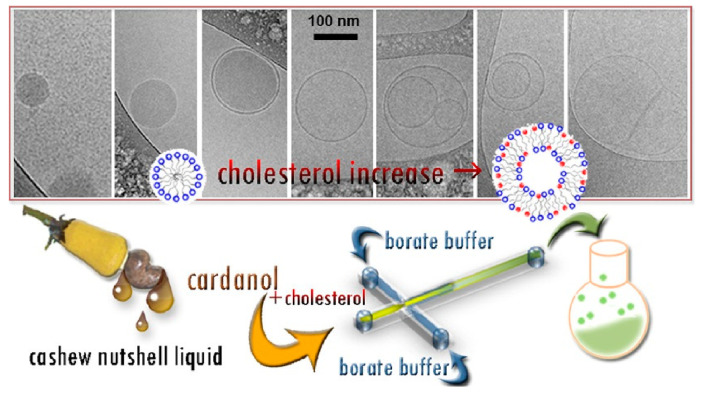
Synthetic scheme of microfluidic preparation of CA-based nanostructures. Reproduced from ref. [35], American Chemical Society, 2022.

A new experimental approach for CA-based amphiphilic nanostructures’ preparation has been carried out via a microfluidic route [35], with the advantage of operating at continuous flows, with a reduced amount of reagents and at lower experimental temperatures, ensuring no waste formation and the achievement of size-monodisperse amphiphilic nanostructures (vesicles up to 100 nm in size) that do not need further purification steps (Figure 8).

Electron microscopy analyses and differential scanning microcalorimetry demonstrate that upon increasing CH in the lipid mixture, a switchover from spherical CA micelles to CA/CH mixed closed vesicles occurs. An improved environmentally friendly process in terms of mildest temperature and pH conditions carried to the preparation of monocomponent nanovesicles, in which synthetic CA-benzoxazines are the sole building blocks of green nanosystems [36]. A green synthesis of CA-benzoxazines via using a choline chloride–urea mixture as a deep eutectic solvent has been carried out in five different formulations. From the results, it can be deduced that the saturated CA-benzoxazine derivative can effectively replace CH as a possible cosurfactant, and the preparation with only the unsaturated BZ derivative can be carried out at milder temperatures, obtaining nanovesicles with a regular spherical shape and smaller dimensions, with good stability.

The following Table 2 summarizes the CA-based nanoformulations discussed so far, providing indications on their potential applications in the technological and electrochemical fields.

Table 3 summarizes the CA-based nanoformulations, providing indications on their potential applications in the biomedical field.

### 2.2. Anacardic Acid-Based Nanomaterials

AA is less popular than CA among researchers because it is obtained from a more expensive treatment of cashew shells, but it is no less attractive. Indeed, it is structurally similar to salicylic acid as a mixture of 2-hydroxy-6-alkylbenzoic acid congeners and has attracted great research interest due to its biological activities [37]. Table 4 summarizes the most recent studies on the tested bioactivity of nanoformulations developed using AA and its derivatives, with the aim of providing a clear and immediate reading of their potential applications in medicine and related fields.

Other researchers, conscious of the well-known potential of AA from both chemical and biological points of view, have focused their studies on the use of AA in the production of nanomaterials. Metallic nanoparticles functionalized with biomolecules have received special attention due to their various biomedical applications. Magnetic iron oxide nanoparticles coated with AA were synthesized and characterized for particle size, magnetic properties, thermal stability, and thermal response in hyperthermia treatments [50]. The AA coating stabilized the nanoparticles by preventing aggregation without losing their magnetization potential. Moreover, the anticancer properties of the AA coating can promote the ability to concentrate at the target area and the stabilization in biological fluids of magnetic nanoparticles, which may be used in a medical application, such as magnetic hyperthermia.

CNSL derivatives, such as AA and CD, form micelles above critical micellar concentration, which can act as passivating agents for silver nanoparticles. Bezerra et al. [51] described the preparation, characterization, and in vitro antileishmanial activity of green-based silver nanoparticles with AA and CD. The synthesis was carried out via reduction with sodium borohydride in the presence of AA or CD under microwave irradiation, obtaining silver nanoparticles with a mean hydrodynamic diameter of 167 nm and 260 nm, respectively. In vitro assays showed opposite effects for AA-Ag nanoparticles and CD-Ag nanoparticles: AA-Ag nanoparticles are highly toxic to macrophages and almost nontoxic for *Leishmania braziliensis*, while CD-Ag nanoparticles are very selective toward killing this last parasite and should be further explored as a promising nontoxic treatment for cutaneous Leishmaniasis.

**Figure 9:**
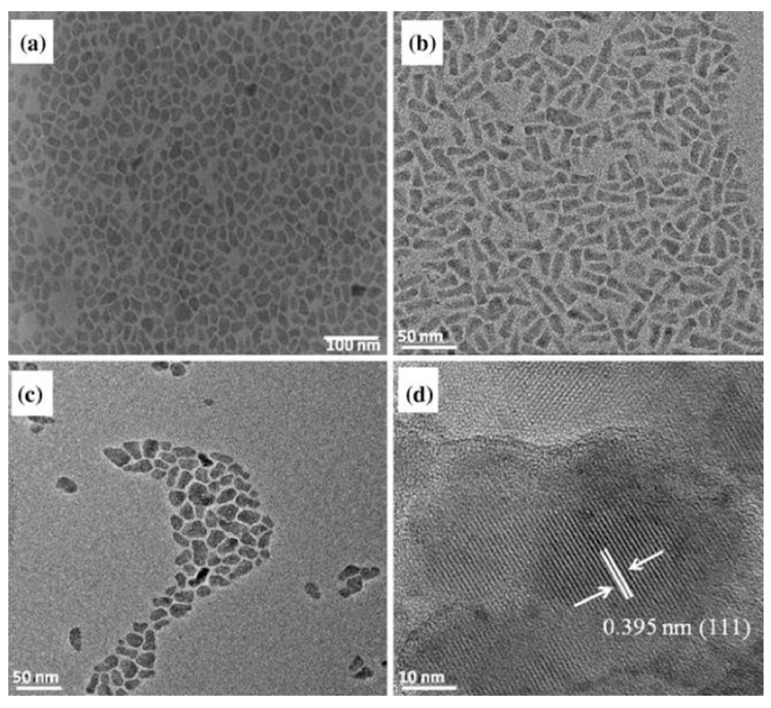
TEM images of AA-capped (**a**) Cd sulfide, (**b**) Cd selenide, and (**c**) Cd telluride, and (**d**) HRTEM image of Cd telluride nanoparticles synthesized at 140 °C. Reproduced from ref. [48], Springer Nature, 2014.

Several studies have exploited the chemical characteristics of AA, rather than its biological properties, to develop nanomaterials with various potential applications. For example, AA was used as a coordinating solvent for the synthesis of metal chalcogenide (Cd and Pb sulfide, selenide, and telluride) nanoparticles via a solution-based technique, obtaining nanoparticles with a well-defined size and morphology [52,53]. TEM analysis (Figure 9) revealed a mosaic-like pattern for all of the samples, and monodispersed spherical to cubic-shaped Pb-chalcogenide nanoparticles were obtained. The optical properties of the AA-capped chalcogenide particles show evidence of quantum confinement, framing them in a class of materials with super-tunable optical and electronic properties suitable for a variety of applications in different fields, such as photovoltaics, light-emitting devices, environmental sciences, and nanomedicine.

An easy way to synthesize biocompatible hybrid silicate nanofibers was proposed by Chacko et al. [54]. Montmorillonite, a self-assembled inorganic–organic silicate hybrid system, was organomodified using cations of aminosilyl derivatives of bio-monomers, such as AA and CA, and showed intercalation attributed to modification via cation exchange and long cis-unsaturated aliphatic chain-induced layer separation. They showed an organization into “arthropodal” branched nanofibers of about 30–60 nm in diameter and 10–20 µm in length, ascribable to rolling into a grain-like morphology stabilized by hydrogen-bonded interactions induced by the carboxyl group and end-to-end assembly of the grains. The absence of any such assembly in the sample modified by cations derived from CA confirmed the carboxyl group effect.

Recently, new formulations following the principles of “green chemistry” have been developed for the preparation of nanovesicles, using AA as the only component or mixed with small amounts of CH [55]. The greater polarity of the AA due to the presence of the carboxylic acid group compared to the CA molecule has allowed the development of different formulations. By varying the preparation conditions, it was possible to evaluate the CH amount reduction effect as well as its complete removal from the AA-based nanostructures. The morphological analysis by TEM reveals that the inclusion of CH doubles the mean diameter of the vesicles to approximately 100 nm, and helps to preserve the initial composition over time, as shown by the stability studies. Instead, the formulation with only AA produced smaller nanovesicles (50 nm) that are rather stable for up to 14 days of storage at 20 °C.

## 3. Conclusions and Perspectives

The increasing use of agro-industry wastes as raw materials has opened a window of opportunity for the development of alternative products to the oil industry. The development of bio-based materials and technologies based on the use of bio-renewable resources represents a strategic approach to offset the economic impact related to the frequent oscillation in petroleum prices. CNSL represents an example of a naturally occurring oil that includes both phenolic and alkenyl structural properties.

These characteristic functional groups can undergo selective or simultaneous chemical modifications by selecting the most suitable chemical approach according to their desirable properties. The present review highlights the potential use of cardanol, anacardic acid, and cardol isolated from a more complex mixture known as CNSL, or natural and technical CNSLs themselves, as prospective “evergreen natural resources” suitable as building blocks to obtain different classes of nanosystems. Recent advances in nanomaterials based on CNSL have been discussed, including their potential applications, among which are biomedical materials and templates for the preparation of nanostructured systems.

## Figures and Tables

**Table 1:** Composition of natural, technical, and distilled CNSL. Prepared based on data from [6,8,9,10].

Cardanol	Anacardic Acid	Cardol	2-Metylcardol	Polymeric Materials	
3–10%	60–70%	10–20%	2–3%	-	Natural CNSL
60–70%	<5%	10–20%	2–3%	7–10%	Technical CNSL
>90%	Not detected	<5%	Not detected	-	Distilled CNSL (180–200 °C)

**Table 2:** Summary of CA-based nanoformulations in technological and electrochemical applications.

Nanoformulation	Technological and Electrochemical Applications	Ref.
ZnO nanostructures impregnated with CA-H_2_Pp and CA-CuPp	Heterogeneous photocatalysts for water purification	[16]
Nano-biocomposites consisting of CA/formaldehyde/epoxy resin polymer added with spongy gourd fiber/magnetite nanoparticles	Dielectric resonators	[19]
Sheet-like nano-biocomposites consisting of CA thermosetting resin/cellulose nanofibrils/expanded graphite nanoplatelets	Coating systems and automotive applications	[20]
CA-benzenesulfonic acid as a dopant for polyaniline nanofibers, nanorods, nanospheres, and nanotubes	Electrochemical properties and processability in electronic and optical devices	[25]
4-(3-dodecyl-8-enylphenyloxy) butane sulfonic acid synthesized by CA as a dopant for polyaniline nanofibers and nanotapes	Bio- and chemical sensors as well as optical devices	[28]

**Table 3:** Summary of CA-based nanoformulations in biomedical applications.

Nanoformulation	Biomedical Applications	Ref.
Nanostructured MOF consisting of CA organic ligand and nontoxic endogenous cation Mn(II) bivalent salt (MnIIMicCol)	Thermally stable antimicrobial coating materials Antibacterial activity against *E. coli*, *K. pneumoniae*, *B. subtilis*, and *S. aureus*	[17]
Core/shell/shell hybrid nanomaterials formed by an Fe_3_O_4_ core/OA layer/CA-Pc layer	Therapeutic agents for photodynamic therapy and magnetic hyperthermia	[18]
Nano-flake fluorescent organogels consisting of pyrene-coupled coumarin derivatives obtained via CA-aldehyde	Inhibitory for the proliferation of PC3 prostate cancer cells Material for cell imaging	[23]
Supramolecular hydrogel from CA-derived coumarin-tris-based amphiphiles	Stimuli-responsive (pH or Fe^3+^ ion) drug delivery system for in vivo formulations	[24]
Nanovesicles from the biobased surfactant N-cardanyl tauramide	Temperature stimuli-responsive drug delivery system	[29]
CA-based nanovesicles via a solvent-free batch method hosting a CA–porphyrin hybrid, chlorogenic acids, phthalazine derivatives, and cannabidiol	Nanosystems for photodynamic therapy, antioxidant and cytotoxic activities against different cancer cell lines, and preserving bioactive embedded molecules	[30,31,32,33]
Fluorescent CA-based nanovesicles via a solvent-free batch method embedded with RhB	Biocompatible green nanotools for bioimaging Active uptake in human macrophages and HeLa cells	[34]
CA-based amphiphilic nanostructures by a microfluidic route (micelles, vesicles)	Bioactive nanosystems for drug delivery	[35]
Nanovesicles via a solvent-free batch method based on CA-benzoxazines synthesized via a green route	Bioactive green nanosystems	[36]

**Table 4:** AA-based nanoformulations in biological applications.

Nanoformulation	Characterization Data	Bioactivity	Ref.
Nanoliposomes based on hydrogenated AA conjugated to a CD133 monoclonal antibody	Size = 100.90 ± 5.24 nm PDI = 0.28 ± 0.02 ZP = −40.7 ± 3.2 mV EE = 100%	Cytotoxic against NTERA-2 cancer stem cells Apoptosis	[38]
Antineoplastic drug: mitoxantrone loaded into liposomal carriers enriched with encapsulated AA in the liposomal bilayer using a vitamin C gradient	Size = 112 ± 3 nm PDI = 0.041 ± 0.003 ZP = −4.31 ± 0.49 mV EE = 99.5 ± 3.5%	Epigenetic agent anticancer	[39]
Liposomes containing AA, mitoxantrone, and ammonium ascorbate	Size = 119 ± 1.5 nm PDI = 0.05 ZP = −3.71 ± 0.5 mV EE = 89.9%	Apoptosis via reactive oxygen species production through the killing of cancer cells in a monolayer culture towards melanoma cells	[40]
AA-loaded zein nanoparticles	Size = 381.6 nm PDI = 0.067 ZP = −15.9 mV AA (%*w*/*v*) = 0.00093	Bacteriostatic for *Staphylococcus aureus* and *Pseudomonas aeruginosa* Bactericide activity for *S. aureus* Fungistatic/fungicide for *Candida rugosa*, *Candida albicans*, *Candida parapsilosis*, *Candida tropicalis*, *Candida jardinii*, *Candida glabratta,* and *Candida auris* Inhibitory/bactericidal for *Planktonic S. mutans* Larvicidal for *A. aegypti larvae*	[41,42,43]
AA-loaded solid lipid nanoparticles, coated with chitosan and DNase	Size = 212.8 ± 4.21 nm PDI = 0.285 ± 0.04 ZP = +13.5 ± 1.92 mV EE = 73.8 ± 1.23%	Antimicrobial efficacy against *Staphylococcus aureus*	[44]
AA, gemcitabine used for the development of docetaxel nanoparticles	Size = 163 ± 8 nm PDI = 0.13 ± 0.09 ZP = −27 ± 1 mV EE = 9.1 ± 0.6%	Tumor targeting through VEGF receptors overexpressed in tumors Combination of gemcitabine and docetaxel provides synergistic activity by targeting multiple pathways	[45]
Docetaxel-loaded AA functionalized liposomes	Size = 126.4 ± 6.2 nm PDI = 0.239 ± 0.03 EE = 72.35 ± 3.46%	Reduction in tumor volume and toxicity in comparison with marketed formulation (Taxotere^®^)	[46]
AA, CD, chitosan, alginate, and gum arabic matrices	Size = 70/250 nm ZP = −18.8/−9.8 mV	Inhibitory capacity for all strains of dermatophytes and antimicrobial control	[47]
AA nanocapsules obtained via interfacial polymerization using the inverse miniemulsion technique with TDI	1 equivalent TDI: Size = 310 ± 17 nm ZP = −34 mV 2 equivalent TDI: Size = 582 ± 153 nm ZP = −43 mV	Active in vitro against *Bacillus subtilis* colonies in the bacterial tests	[48]
Self-aggregated nanoparticles from chitosan modified with AA	Size = 214 nm ZP = −20.3 mV EE = 27 ± 3%	Release and stabilization of insulin in the intestinal environment	[49]

## Data Availability

Not applicable.

## Abbreviations

CNSL	Cashew nut shell liquid
CA	Cardanol
AA	Anacardic acid
CD	Cardol
CH	Cholesterol
O/W	Oil/water
tCNSL	Technical CNSL.
H_2_Pp	Metal-free porphyrin.
CuPp	Copper porphyrin.
Pc	Phthalocyanine.
OA	Oleic acid.
FTIR	Fourier-transform infrared spectroscopy.
UV	Ultraviolet.
TGA	Thermogravimetric analysis.
DSC	Differential scanning calorimetry.
DTG	Derivative thermogravimetry.
XRD	X-ray diffraction.
TEM	Transmission electron microscopy.
SEM	Scanning electron microscopy.
RhB	Rhodamine B.
VOCs	Volatile organic compounds.
MOF	Metal–organic framework.
TEM	Transmission electron microscopy.
HRTEM	High resolution transmission electron microscopy.
^1^HNMR	Proton nuclear magnetic resonance.
DLS	Dynamic light scattering.
PDI	Polydispersity index.
ZP	Zeta potential.
EE	Encapsulation efficiency.
DNase	Deoxyribonuclease I.
TDI	Toluene 2,4-diisocyanate.
